# Sodium–Glucose Cotransporter 2 Inhibitors and Kidney Outcomes: True Renoprotection, Loss of Muscle Mass or Both?

**DOI:** 10.3390/jcm9051603

**Published:** 2020-05-25

**Authors:** Adrian Post, Dion Groothof, Michele F. Eisenga, Stephan J. L. Bakker

**Affiliations:** Department of Nephrology, University Medical Center Groningen, University of Groningen, 9713 GZ Groningen, The Netherlands; d.groothof@umcg.nl (D.G.); m.f.eisenga@umcg.nl (M.F.E.); s.j.l.bakker@umcg.nl (S.J.L.B.)

**Keywords:** SGLT2 inhibitors, muscle mass, eGFR, renoprotection

## Abstract

Inhibitors of sodium–glucose cotransporter 2 (SGLT2) have emerged as practice-changing treatments for patients with type 2 diabetes, reducing both the risk of cardiovascular events and kidney events. However, regarding the latter, caution is warranted, as these kidney endpoints are defined using glomerular filtration rate estimations based on creatinine, the non-enzymatic product of creatine residing in muscles. Creatinine-based estimations of the glomerular filtration rate are only valid if the treatment has no effect on changes in muscle mass over time. Yet, circumstantial evidence suggests that treatment with SGLT2 inhibitors does result in a loss of muscle mass, rendering serum creatinine-based kidney endpoints invalid. Currently, it cannot be excluded that the described renoprotective effect of SGLT2 inhibitors is in part or in whole the consequence of a loss of muscle mass. Post-hoc analyses of existing trials or new trials based on kidney function markers independent of muscle mass can provide more definitive answers on the proposed renoprotective effects of SGLT2 inhibitors.

## 1. Introduction

Diabetes mellitus is estimated to affect more than 450 million people worldwide and expectations suggest this number to further increase to 690 million by 2045 [1]. Approximately 40% of them will eventually develop diabetic nephropathy, posing diabetic nephropathy as the leading cause of end-stage kidney disease (ESKD) [2]. Along with the associated high morbidity and mortality rates, it constitutes an enormous public health burden.

The introduction of angiotensin-converting enzyme inhibitors as renoprotective treatment, followed by angiotensin II receptor blockers signified important steps forward in the secondary prevention of diabetic nephropathy [3,4,5]. Nevertheless, the global health burden has increased incessantly, igniting the need for additional treatment alternatives. After years of draught and failure with many first seemingly promising treatments (e.g., dual RAAS blockade, thiazolidinediones, bardoxolone methyl), the sodium-glucose cotransporter 2 (SGLT2) inhibitors arose as a new promising treatment option [6]. SGLT2 inhibitors were initially approved as a new class of glucose-lowering agents in patients with type 2 diabetes enhancing the urinary glucose excretion through the inhibition of glucose reabsorption in the proximal convoluted tubule.

## 2. SGLT2 Inhibitors and Kidney Events

Since 2008, the FDA has demanded that all new glucose-lowering agents undergo long-term cardiovascular outcome trials. In these trials, SGLT2 inhibitors exhibited significant reductions in the risk of atherosclerotic cardiovascular events and heart failure-related hospitalizations [7,8,9]. Furthermore, post-hoc analyses suggested strong renoprotective effects of SGLT2 inhibitor treatments (EMPA-REG OUTCOME, CANVAS Program) [10,11]. Importantly, this was against a background of angiotensin-converting enzyme inhibitors and angiotensin II receptor blockers, so it would be renoprotection on top of existing treatment. Most participants, however, were at low risk of ESKD and the effect of SGLT2 inhibitors on the need for dialysis or transplantation was therefore uncertain. The recently conducted Canagliflozin and Renal Events in Diabetes with Established Nephropathy Clinical Evaluation (CREDENCE) trial [12]—specifically designed to address this lacuna—suggests that canagliflozin substantially reduces the risk of incident kidney failure. Furthermore, compared to the placebo group, patients treated with canagliflozin exhibited sizable reductions in glycated haemoglobin, blood pressure, body weight, albuminuria, and the slope of kidney function deterioration. These findings were recently corroborated by a meta-analysis investigating the effects of SGLT2 inhibitors on kidney outcomes [13].

Notwithstanding these findings, some caution is warranted before SGLT2 inhibitors become mainstay in treatment of diabetic nephropathy—and potentially in prevention of kidney function deterioration in any kidney disease associated with glomerular hyperfiltration. The reason being that the EMPA-REG OUTCOME, CANVAS, and CREDENCE trials do not unequivocally provide evidence for renoprotection.

In each of the aforementioned trials, the kidney endpoints rest upon serum creatinine-based estimations of the glomerular filtration rate (GFR), which applies to both the endpoint of rate of change in estimated GFR (eGFR) and the endpoint of initiation of dialysis or transplantation [14]. Creatinine is a non-enzymatic degradation product of the creatine pools, which reside primarily in muscle tissue. Therefore, endpoints relying on creatinine-based estimations of the glomerular filtration rate are only valid if the treatment has no effect on changes in muscle mass over time. However, the following circumstantial evidence suggests that treatment with SGLT2 inhibitors does result in loss of muscle mass.

## 3. Effects of SGLT2 Inhibitors on Muscle Mass

SGLT2 inhibitors intrinsically reduce the insulin to glucagon ratio through their pharmacological mechanisms of action, thereby serving as a stimulant for hepatic gluconeogenesis [15], and as soon as glucose molecules excreted in urine are derived from gluconeogenesis, this will inevitably lead to loss of both fat and muscle mass. Studies reporting on changes in body weight during SGLT2 inhibitor treatment have unequivocally shown weight reductions during SGLT2 inhibitor treatment, a topic extensively reviewed by Pereira et al. [16] and Lee et al. [17]. Network meta-analyses estimated the reductions in body weight to be about 1.5 to 2 kg (kg) compared with placebo for all SGLT2 inhibitors [18,19,20], wherein the degree of weight loss depended on the drug and dosage (e.g., 1.6 kg of weight reduction for dapagliflozin 5 mg and 2.5 kg for canagliflozin 300 mg [19]). Since it is unlikely that SGLT2 inhibitor treatment will lead to increased physical activity, this can best be compared to weight loss induced by dietary restrictions, an effect known to be accompanied by both loss of fat mass and loss of muscle mass [21]. In addition, part of the weight loss of SGLT2 inhibitors may be due to a loss of body water together with sodium. Several studies found no effect of treatment on muscle mass [22,23,24,25], but the results of many other studies are consistent with a reduction in both fat tissue and muscle tissue [26,27,28,29,30,31,32]. Though most of the weight loss during treatment usually is the consequence of loss of fat mass, a relatively low loss of muscle mass can already be enough to materially influence trajectories of eGFR, if based on serum creatinine (see our calculations in Box 1). A single-arm study investigating the effects of 12-week tofogliflozin (20 mg/day) in 37 patients with type 2 diabetes on body composition showed 0.8 kg reduction in skeletal muscle mass, which corresponds to a 2.8% change from baseline [32]. Similarly, another single-arm study investigating the effects of 12-week add-on tofogliflozin (20 mg/day) to DPP-4 inhibitor treatment in 16 patients with type 2 diabetes demonstrated a muscle mass reduction of 1.4 kg [31]. Comparable changes were found after 24 weeks of treatment with ipragliflozin (50 mg/day) in 20 patients with type 2 diabetes [30]. Using dual-energy X-ray absorptiometry, a 1.7 kg reduction in lean body mass and a 0.6 kg reduction in appendicular lean mass were observed after 24 weeks, corresponding to a 3.3% and 2.8% change from baseline, respectively [30]. In the LIGHT trial, luseogliflozin (2.5 mg/day) led to a 1.0 kg reduction in lean body mass after 52 weeks in 37 patients with type 2 diabetes [29], corresponding to a 2.3% change from baseline lean body mass. Unlike these single-arm studies, the CANTATA-SU trial included 1450 patients with type 2 diabetes, who were randomly assigned (1:1:1) to either canagliflozin 100 mg, canagliflozin 300 mg, or glimepiride [28]. After 52 weeks, patients receiving canagliflozin 100 mg and canagliflozin 300 mg displayed a 0.9 kg and 1.1 kg reduction in lean body mass, corresponding to a 1.8% and 2.5% change from baseline lean body mass, respectively [28]. Compared to glimepiride, the mean reductions in lean body mass were 2.0 kg and 2.2 kg for canagliflozin 100 mg and canagliflozin 300 mg, corresponding to reductions of 4.2% and 4.9% compared to baseline, respectively [28]. Another study investigating the effects of canagliflozin on body composition also found reductions in lean body mass [27]. In this study, 714 patients with type 2 diabetes were randomized to placebo, canagliflozin 100 mg, or canagliflozin 300 mg for 26 weeks [27]. Compared to placebo arm, patients on canagliflozin 100 mg lost 0.6 kg lean body mass and patients on canagliflozin 300 mg arm lost 0.9 kg lean body mass, corresponding to a 1.2% and 1.7% change from baseline lean body mass, respectively [27]. These results were corroborated by single-arm study showing a 1.1 kg decrease in lean body mass after 24 weeks of canagliflozin treatment (100 mg/d), corresponding to a 2.2% decrease from baseline [26]. An overview of muscle mass changes during SGLT2 inhibitor therapy is demonstrated in Table 1. In summary, a variety of studies investigating the effect of SGLT2 inhibitors on body composition demonstrated that SGLT2 inhibitor therapy is associated with a significant reduction in muscle mass.

Box 1 **Simplified calculation explaining the effect of serum creatinine reductions, reflecting loss of muscle mass, on changes in estimated GFR over time.**

eGFR = 141 ∗ (Scr/0.9)^−1.209^ ∗ 0.993^Age^ (1)

ΔeGFR = eGFR_year x+1_ − eGFR _year x_ (2)

ΔeGFR = (141 ∗ (Scr _year x+1_/0.9)^−1.209^ ∗ 0.993^Age+1^) − (141 ∗ (Scr _year x_/0.9)^−1.209^ ∗ 0.993^Age^) (3)

ΔeGFR = (141 ∗ (Scr_year x+1_/0.9)^−1.209^ ∗ 0.993^64^) − (141 ∗ (1.34/0.9)^−1.209^ ∗ 0.993^63^) (4)

ΔeGFR = (89.9 ∗ (Scr_year x+1_/0.9)^−1.209^) − 56.0 (5)

89.9 × (Scr_year x+1_/0.9)^−1.209^ = ΔeGFR + 56.0 (6)

(Scr_year x+1_/0.9)^−1.209^ = (ΔeGFR + 56.0)/89.9 (7)

Scr_year x+1_/0.9 = ((ΔeGFR + 56.0)/89.9)^−0.827^ (8)

Scr_year x+1_ = 0.9 ∗ ((ΔeGFR + 56.0)/89.9)^−0.827^ (9)

Scr_year x+1_ = 0.9 ∗ ((1.52 + 56.0)/89.9)^−0.827^   Scr_year x+1_ = 0.9 ∗ ((2.74 + 56.0)/89.9)^−0.827^ (10)

Scr_year x+1_ = 1.30 mg/dL      Scr_year x+1_ = 1.28 mg/dL (11)

Change (%) = (1.30 − 1.34)/1.34 ∗ 100%   Change (%) = (1.28 − 1.34)/1.34 ∗ 100%(12)

Change (%) = −2.8%      Change (%) = −4.5%(13)

The change in serum creatinine required to explain a certain yearly difference in kidney function deterioration, between the canagliflozin and placebo group, can be calculated. For these calculations, we used a hypothetical example, based on the average age and eGFR at baseline of the CREDENCE trial. Therefore, calculations are performed for a white, 63-year-old, male subject, with an eGFR of 56 mL per minute per 1.73 m^2^—a value which corresponds to a serum creatinine of 1.34 mg/dL [37]. In this example, we aim to determine what change in serum creatinine would be required to explain the between-group difference in eGFR slopes between the canagliflozin and placebo group of the CREDENCE trial by the relative decline in muscle mass, rather than by the relative improvement in renal function. To simplify calculations, the calculations are performed for a timeframe of one year and we use a delta eGFR (ΔeGFR) rather than a slope. ΔeGFR is defined as the difference between eGFR at timepoint x and timepoint x + 1 year (formulas nr. 1–3) and is used to resemble the between-group difference in eGFR slopes between the canagliflozin and placebo group of the CREDENCE trial. For our calculations we define the age at timepoint x as 63 years and serum creatinine at timepoint x as 1.34 mg/dL (formulas nr. 4–5). The equation is rewritten to calculate the serum creatinine value after a year (formulas nr. 6–9), assuming a certain ΔeGFR. The formula now is written so that it can be used to calculate the serum creatinine value after one year that is required to fully explain a certain ΔeGFR.

In the CREDENCE trial, the least-squares mean change in the eGFR slope was less in the canagliflozin group than in the placebo group (–3.19 ± 0.15 vs. –4.71 ± 0.15 mL per minute per 1.73 m^2^ per year). This corresponds to a between-group difference of 1.52 mL per minute per 1.73 m^2^ per year. If we normalize the eGFR slope of the canagliflozin group to the placebo group, this indicates a yearly relative increase in eGFR of 1.52 mL per minute per 1.73 m^2^. Therefore, we can substitute ΔeGFR for this value and use the formula to calculate what decrease in serum creatinine as a consequence of a putative decrease in muscle mass would be able to fully explain this difference in slope (formulas nr. 10–11). These calculations show that a 2.8% decrease in serum creatinine (from 1.34 to 1.30 mg/dL in the first year of the trial) is enough to fully explain a 1.52 mL per minute per 1.73 m^2^ difference in kidney function deterioration between the canagliflozin and placebo group (formulas nr. 12–13) during the first year of the trial and an additional 2.8% per year in each following years. Because there is a proportional relationship between muscle mass and serum creatinine, this indicates that an average yearly loss of 2.8% of muscle mass during the years of the trial can explain the seeming average of 1.52 mL per minute per 1.73 m^2^ less deterioration in eGFR. This means that, for each year of the trial, even if there was no actual renoprotection, but rather a yearly loss of muscle mass of 2.8%, a seeming difference of 1.52 mL per minute per 1.73 m^2^ per year (compared to the placebo group) could be explained.

Because in the CREDENCE trial a large drop in eGFR in response to the start of canagliflozin treatment compared to placebo was observed during the first 3 weeks—which cannot be explained by effects on muscle mass, but is very likely due to mitigation of renal hyperfiltration by canagliflozin—we also performed alternative calculations based on reported differences in eGFR slopes between the canagliflozin and placebo group, in which the effects on eGFR during the first three weeks of the trial were discarded. After excluding the effects of canagliflozin and placebo on eGFR during the first three weeks of the trial, the CREDENCE investigators reported an average between-group difference in the slope of eGFR of 2.74 mL per minute per 1.73 m^2^ per year. If we use this value instead of the value of 1.52 mL per minute per 1.73 m^2^ per year, a 4.5% decrease in serum creatinine (from 1.34 mg/dL to 1.28 mg/dL in the first year of the trial, if the effects on eGFR observed during the first three weeks are adjusted for) would be required to fully explain a difference of 2.74 mL per minute per 1.73 m^2^ during each year of the trial.

## 4. The Effect of Loss of Muscle Mass on Creatinine-Based eGFR Trajectories

The results of the aforementioned studies fuel the plausibility that treatment with SGLT2 inhibitors will result in a loss of muscle mass and, hence, a loss of the endogenous creatine pool from which creatinine is synthesized. On a daily basis, roughly 1.7% of the total creatine pool is converted to creatinine through a low-grade non-enzymatic degradation reaction [45]. A decrease in muscle mass therefore implicates a decrease in serum creatinine, which, in turn, steers towards to an underestimation of kidney function trajectories when such estimates are derived from serum creatinine concentrations. Compared to a stable muscle mass over time, a loss of muscle mass over time erroneously suggests either improvement in kidney function or slower rate of kidney function deterioration.

In the CREDENCE trial, the difference in annual deterioration of eGFR between the SGTL2 inhibitor group and the placebo group was 1.52 mL per minute per 1.73 m^2^. Because of the proportional relationship between muscle mass and serum creatinine, a similar difference could be achieved by a 2.8% reduction in muscle mass in a 63-year-old white male population with a baseline eGFR of 56 mL per minute per 1.73 m^2^ (Box 1). This percentage reduction in muscle mass is comparable to the relative changes in lean body mass and/or muscle mass observed in studies investigating the effects of SGLT2 inhibitors on body composition, i.e., 2.5% lean body mass reduction (4.9% when compared to the control group) after 52 weeks of canagliflozin (300 mg/day) treatment in the CANTATA-SU trial [28]. It should be noted, however, that during the first 3 weeks of the CREDENCE trial, a greater reduction in the eGFR was observed in the canagliflozin group compared with the placebo group. After excluding the first three weeks, the annual deterioration in kidney function between the SGTL2 inhibitor group and the placebo group was 2.74 mL per minute per 1.73 m^2^. A similar annual difference could be achieved by a 4.5% reduction in muscle mass per year (Box 1).

It is important to generate investigational data to confirm or not confirm this hypothesized scenario, since continued loss of muscle mass will comprise patients’ capability to recover from intercurrent illnesses with increased risk of premature mortality [46,47,48]. If confirmation was the case, it could even be hypothesized that protective effects of SGLT-2 inhibitors in relatively short running long-term outcome trials could turn into adverse effects if trial periods were extended from a typical period of 3 years to, e.g., 10 years.

Importantly, it is not only the endpoint of rate of change in eGFR which is affected by muscle mass. It is likely that the endpoint of initiation of dialysis or transplantation is affected by changes in muscle mass as well, since eGFR also plays a role in decisions to start with dialysis or to proceed with transplantation [49]. Treatment regimens inducing loss of muscle mass will, by consequence, cause an overestimation of eGFR and hence a deferral in the initiation of dialysis or transplantation when such estimates are based on serum creatinine. Furthermore, the diuretic nature of SGLT2 inhibitors will intrinsically prevent fluid overload—a common cause for initiating dialysis [50]—further driving the result towards the seeming prevention of the endpoint of initiation of dialysis or transplantation.

We therefore hypothesize that the prevention of kidney function deterioration—as surmised in the EMPA-REG OUTCOME, CANVAS, and CREDENCE trials—may at least partially be the consequence of a treatment-induced loss of muscle mass. It would be of substantial interest to investigate whether long-term SGLT2 inhibitor treatment truly inflicts loss of muscle mass. If so, we propose that the potential renoprotective effects of SGLT2 inhibitors be thoroughly analyzed with muscle mass independent measures of GFR. Muscle mass independent measures of GFR include estimation of the GFR based on cystatin C or symmetric dimethylarginine. Alternatively, the glomerular filtration rate can be measured by direct methods—e.g., by the assessment of inulin clearance—although these methods are more invasive and time-consuming than the muscle mass independent methods for the estimation of GFR.

It should be noted that our discussion and hypothesis apply to long-term changes in eGFR, and not to changes in albuminuria. There is convincing evidence that SGLT2 inhibitors protect against the development of albuminuria [51], which is an acknowledged risk factor for development of ESKD, with meta-analyses showing that a 30% reduction in albuminuria is associated with a 24% reduction in the risk of development of ESKD [52].

Another noteworthy limitation of many of the studies that have investigated changes in body composition during SGLT2 inhibitor therapy comprises their use of measurements of lean body mass, rather than specific components making up lean body mass. Lean body mass does not equal muscle mass, but also bone, skin, and other organs. More importantly, lean body weight and muscle mass are also influenced by hydration status, while in an ideal situation, one would like to have information on dry fat mass and dry muscle mass. Additionally, it should be noted that most of the studies investigating the effect of SGLT2 inhibitors on body composition have used bioimpedance or dual-energy x-ray absorptiometry, and only a few have used other techniques, including magnetic resonance imaging, computerized tomography, or the urinary creatinine excretion rate. Lastly, it is important to note that the precision of the serum creatinine measurements was not discussed in this perspective, though reported inter-assay coefficients of variation of up to 2.65% may hinder detection of relatively small differences in serum creatinine over time in individual subjects [53,54]. 

## 5. Conclusions

Overall, SGLT2 inhibitors have emerged as practice-changing treatments for patients with type 2 diabetes. However, regarding the proposed effects on eGFR based kidney events, some caution is warranted. There are reasons to believe that SGLT2 inhibitor treatment results in the loss of muscle mass over time, thereby rendering serum creatinine-based kidney endpoints invalid. Therefore, at this time, it cannot be excluded that the described renoprotective effect of SGLT2 inhibitors is in whole or in part the consequence of a loss of muscle mass. Post-hoc analyses of existing trials or new trials based on measures of GFR independent of muscle mass can provide more definitive answers on the proposed renoprotective effects of SGLT2 inhibitors.

## Figures and Tables

**Table 1:** Overview of the effects of SGLT2 inhibitors on lean body mass and muscle mass.

Study	SGLT2 Inhibitor	Dosage (mg/Day)	Time (Weeks)	Design	Participants	Measurement	Technique	Baseline Value	Change *	Significance	Percentual Change **
**Canagliflozin**											
Blonde et al. [27]	Canagliflozin	100 300	26	Double-blind randomized placebo controlled parallel group	166	Lean body mass	DXA	51.2 kg 53.2 kg	−0.6 kg −0.9 kg	Yes	−1.2% (−2.4%) −1.7% (−3.4%)
Cefalu et al. [28]	Canagliflozin	100 300	52	Double-blind randomized, active controlled parallel group	208	Lean body mass	DXA	47.7 kg 44.6 kg	−2.0 kg −2.2 kg	Yes	−4.2% (−4.2%) −4.9% (−4.9%)
Koike et al. [26]	Canagliflozin	100	24	Single-arm open-label	38	Lean body mass	DXA	49.6 kg	−1.1 kg	Yes	−2.2% (−4.8%)
Inoue et al. [33]	Canagliflozin	100	52	Single-arm open-label	20	Lean body mass	BIA	51.5 kg	−0.2 kg	No	−0.4% (−0.4%)
Seko et al. [34]	Canagliflozin and Ipragliflozin	100 _(Cana)_ 50 _(Ipra)_	24	Single-arm open-label	24	Skeletal muscle mass	BIA	25.4 kg	−0.6 kg	Yes	−2.3% (−5.1%)
**Ipragliflozin**											
Inoue et al. [24]	Ipragliflozin	50	24	Open-label randomized controlled parallel group	49	Muscle mass and lean mass	BIA DXA	47.1 kg 41.0 kg	−0.38 kg −0.60 kg	No No	−0.8% (−1.7%) −1.5% (−3.2%)
Ohta et al. [30]	Ipragliflozin	50	24	Single-arm open-label	20	Lean body mass and appendicular lean mass	DXA	52.2 kg 21.8 kg	−1.7 kg −0.6 kg	Yes Yes	−3.3% (−7.1%) −2.7% (−6.0%)
Kato et al. [35]	Ipragliflozin	50	12	Single-arm open-label	20	Muscle mass	BIA	n.r.	−0.92 kg	Yes	n.a
Miyake et al. [36]	Ipragliflozin	50	24	Single-arm open-label	12	Skeletal muscle mass	BIA	22.75 kg	−0.50 kg	No	−2.2% (−4.8%)
Yamamoto et al. [37]	Ipragliflozin	50	16	Single-arm open-label	24	Skeletal muscle index	BIA	7.5 kg/m^2^	−0.2 kg/m^2^	Yes	−2.7% (8.7%)
**Luseogliflozin**											
Bouchi et al. [38]	Luseogliflozin	2.5 to 5	12	Single-arm open-label	19	Skeletal muscle index	DXA	7.81 kg/m^2^	−0.23 kg/m^2^	Yes	−2.9% (−12.8%)
Seino et al. [39]	Luseogliflozin	2.5 to 5	52	Single-arm open-label	22	Lean body mass	BIA	45.25 kg	−0.44 kg	No	−1.0% (−1.0%)
Sasaki et al. [29]	Luseogliflozin	2.5 to 5	52	Single-arm open-label	36	Skeletal muscle mass index	DXA	7.74 kg/m^2^	−0.155 kg/m^2^	Yes	−2.0% (−2.0%)
**Dapagliflozin**											
Bolinder et al. [40]	Dapagliflozin	10	24	Double-blind randomized placebo controlled parallel group	182	Lean body mass	DXA	56.2 kg	−0.60 kg	Yes	−1.1% (−2.3%)
Kosugi et al. [41]	Dapagliflozin	5	12	Single-arm open-label	26	Lean body mass	DXA	52.0 kg	−0.50 kg	No	−1.0% (−4.2%)
Fadini et al. [42]	Dapagliflozin	10		Single-blind placebo controlled parallel group	31	Lean body mass	BIA	n.r.	−2.9 kg	Yes	n.a.
Tobita et al.	Dapagliflozin	5	24	Single-arm open-label	11	Skeletal muscle mass	BIA	24.6 kg	+0.1 kg	No	+0.4% (+0.9%)
Lundkvist et al. [25]	Dapagliflozin	10	24	Double-blind randomized placebo controlled parallel group	50	Total lean tissue	MRI	42.6 L	−0.19 L	No	−0.4% (−1.0%)
Sugiyama et al. [23]	Dapagliflozin	5	26	Open-label active controlled parallel group	50	Skeletal muscle mass	BIA	28.7 kg	−0.2 kg	No	−0.7% (−1.4%)
**Tofogliflozine**											
Kamei et al. [32]	Tofogliflozin	20	12	Retrospective single-arm open-label	37	Muscle mass	BIA	29.8 kg	−0.8 kg	Yes	−2.7% (−11.6%)
Matsuba et al. [31]	Tofogliflozin	20	12	Single-arm open-label study	16	Muscle mass	BIA	n.r.	−1.37 kg	Yes	n.a.
Iwahashi et al. [43]	Tofogliflozin	20	48	Single-arm open-label study	20	Lean body mass	BIA	47.3 kg	+0.2 kg	No	+0.4% (+0.5%)
**Empagliflozin**											
Javed et al. [44]	Empagliflozin	25	12	Open-label randomized placebo controlled parallel group	39	Lean body mass	BIA	54.8 kg	−1.7 kg	Yes	−3.1% (−13.4%)

Abbreviations: n.r.: not reported; n.a.: not available; BIA: bioelectrical impedance; DXA: dual-energy x-ray absorptiometry. * For studies with a control group defined as the difference in change vs. the control group. ** Percentage change during the study period is given first and the corresponding percentage change calculated for one year if change in muscle mass would proceed at the same pace is given between brackets.
